# Clinical and Immunological Features of a Large DiGeorge Syndrome Cohort

**DOI:** 10.1007/s10875-025-01884-0

**Published:** 2025-06-03

**Authors:** Merve Süleyman, Deniz Cagdas, Pelin Özlem Şimşek Kiper, Gülen Eda Ütine, Merve Kaşıkcı Çavdar, Feyzi İlhan Tezcan

**Affiliations:** 1 Department of Pediatrics, Hacettepe University Medical Faculty, Ankara, Turkey; 2 Ihsan Dogramaci Children`s Hospital, Hacettepe University Medical Faculty, Ankara, Turkey; 3 Division of Pediatric Immunology, Department of Pediatrics, Hacettepe University Medical Faculty, Ankara, Turkey; 4 Division of Immunology, Department of Pediatric Basic Sciences, Institute of Child Health, Hacettepe University, Ankara, Turkey; 5 Division of Pediatric Genetics, Department of Pediatrics, Hacettepe University Medical Faculty, Ankara, Turkey; 6 Division of Genetics, Department of Pediatric Basic Sciences, Institute of Child Health, Hacettepe University, Ankara, Turkey; 7 Department of Biostatistics, Hacettepe University Medical Faculty, Ankara, Turkey

**Keywords:** Defects in thymic development, DiGeorge syndrome, 22q11.2 deletion, increased NK, increased CD8, immunodeficiency

## Abstract

**Background:**

DiGeorge Syndrome (DGS), a microdeletion syndrome, shows a broad spectrum from mild T-cell lymphopenia to severe combined immunodeficiency.

**Aim:**

To define the clinical/immunophenotypical biomarkers for DGS.

**Patients and Methods:**

A total of 72 patients with 22q11.2 deletion(*n* = 66) and those fulfilling the DGS criteria without deletion (*n* = 6) were enrolled.

**Results:**

The male/female ratio was 41/31. Median age at clinical diagnosis was 1.7 years (0 days-22 years) with follow-up for 21.7 months (0 days-17.3 years). Common evaluation reasons were cardiac features (30.6%), failure to thrive (15.3%), and neurological features (15.3%). Craniofacial dysmorphism (64/66, 97%), central nervous system findings (62/72, 86.1%), and congenital cardiovascular defect (43/70, 61.4%) were common. We noted lymphopenia (30/72, 41.7%) and low IgM (25/69, 36.2%). T helper cell counts were low in 49.3% (33/67). T cytotoxic and NK cell counts were normal/high in 80.6% (54/67) and 97% (65/67) of patients, respectively. 42.3% (11/26) had low CD4 + TEMRA, and 34.6% (9/26) had low CD8 + TEM percentages. None had low CD8 + TEMRA. B cells were normal/high (52/67, 77.6%). 30.8%(8/26) had low switched-memory and 38.5% (10/26) had low active B cell percentages. Low IgA levels were associated with decreased lymphocyte activation and recent thymic emigrant (RTE) cell percentages. Six(8.3%) patients with lymphopenia, three of whom had congenital athymia, died.

**Conclusion:**

CD4 lymphopenia was more common than CD8 lymphopenia. Normal/high CD8 + and NK cell counts were remarkable. Increased CD8+ TEMRA cells seem to indicate peripheral homeostatic proliferation following viral infections. Low serum IgA correlated with low RTE% and impaired T-cell function. DGS severity markers include hypocalcemia, congenital cardiac anomaly, and T-cell lymphopenia.

**Graphical Abstract:**

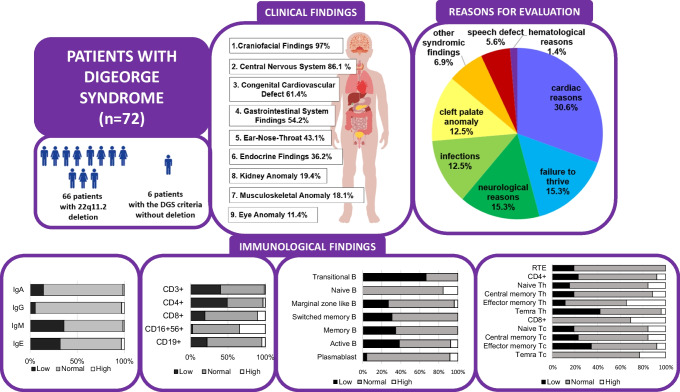

**Supplementary Information:**

The online version contains supplementary material available at 10.1007/s10875-025-01884-0.

## Introduction

The thymus is essential for T-cell development [1]. The most common defect that affects thymic development is an approximately 3-megabase deletion in the 22q11.2 chromosomal region [2]. Defects in thymic development (DTD) is a common name for 22q11.2 deletion syndrome (DS), numerous defects that cause DiGeorge Syndrome (DGS), and for other diseases with thymic defects, such as CHARGE (coloboma, heart defect, atresia choanae, retardation of growth and development, genital hypoplasia, ear anomalies/deafness) syndrome [1]. *CHD7, TBX1, FOXN1, FOXI3, PAX1, TBX2* gene mutations, diabetic embryopathy, retinoic acid embryopathy, and maternal alcohol consumption are other well-known causes of DTD [1].

Phenotypic findings and immune system involvement show a spectrum of 22q11.2 DS [3]. The humoral immune status depends on the degree of T-cell deficiency [4]. Low immunoglobulin levels are frequent in DGS/22q11.2 DS/DTD, and low IgM and IgA levels are generally observed [5].

Complete DGS or DGS patients with congenital athymia have severe immunodeficiency, and other DGS patients have mild to moderate T-cell lymphopenia [6]. B and NK (CD16+56+) cell counts are usually high or normal [4, 7, 8].

Diagnosis of inborn errors of immunity (IEI) is often late, essential treatment and management cannot be implemented on time, and recurrent infections and complications lead to increased morbidity and mortality. Although the DGS/22q11.2 DS/DTD spectrum is relatively common in childhood, it may take time to diagnose patients with milder and subtle manifestations, especially in countries where neonatal screening tests for immunodeficiency diseases, such as TREC and KREC are not available. In this study, we aimed to investigate clinical findings, course, and genetic, phenotypic, and immunological characteristics of DGS.

## Patients and Methods

We reviewed 100 DGS patients followed at Hacettepe University Ihsan Doğramacı Children's Hospital between January 2000 and May 2019. We excluded 28, seven patients without a 22q11.2 deletion or not meeting the European Society for Immunodeficiencies (ESID) DGS criteria and 21 with missing clinical data (https://esid.org/Working-Parties/Clinical-Working-Party/Resources/Diagnostic-criteria-for-PID2#Q5). We included 72 patients in the study (Figure S1). Overall, the study group included patients with 22q11.2 deletion and individuals who met the DGS diagnostic criteria.

We analyzed the patient files and data from the hospital information system between March 2019 and May 2020. We recorded patient demographic characteristics, including sex and age at diagnosis, clinical findings, laboratory findings, genetic results, treatments, and survival data. We defined the age at diagnosis as the age at genetic diagnosis for patients with 22q11.2 deletions and the age at the clinical diagnosis for patients without 22q11.2 deletions. We recorded the period from the first complaints to DGS diagnosis as the diagnostic delay and that from the first to last visit to the hospital (time of death for patients who died) as the follow-up period. We used the Turkish version of the Denver Developmental Screening Test II results for the neurodevelopmental evaluation of patients [9]. A single pediatric cardiologist (SNS) reevaluated the cardiovascular findings. Table 1 summarizes the systemic involvement in the given number of patients.

**Table 1:** Systemic involvement in DGS patients

	Patients evaluated	Patients with positive findings		Patients evaluated	Patients with positive findings
	**n**	**n (%)**		**n**	**n (%)**
**Craniofacial Findings**	66	64 (97)	**Other System Findings**		
Auricular deformity	63	51 (80.9)	**Central Nervous System Findings**	72	62 (86.1)
Microretrognathia	66	40 (60.6)	Developmental delay	70	53 (75.7)
Ocular abnormalities	65	29 (44.6)	Special education status	67	44 (65.7)
Depressed nasal bridge, tubular or bulbous nose	66	27 (40.9)	Seizure	71	28 (39.4)
Cleft palate	71	17 (23.9)	CNS malformation	21	11 (52.4)
**Congenital Cardiovascular Defect**	70	43 (61.4)	Attention deficit hyperactivity disorder	64	10 (15.6)
Conotruncal cardiac anomaly	43	17 (39.5)	**Ear—Nose—Throat**	**72**	**31 (43.1)**
*Tetralogy of Fallot*	43	7 (16.3)	Nasal speech	57	20 (35.1)
*Aortic arch anomalies*	43	6 (14)	Cleft palate	71	17 (23.9)
*-Interrupted aortic arch*	43	3 (7.0)	Hearing loss	70	10 (14.2)
*-Vascular ring*	43	2 (4.7)	Cleft lip	71	2 (2.8)
*-Coarctation of the aorta*	43	1 (2.3)	**Gastrointestinal System Findings**	72	39 (54.2)
*Pulmonary atresia* and *ventricular septal defect*	43	2 (4.7)	Malnutrition	67	20 (29.9)
*Truncus arteriosus* and *interrupted aortic arch*	43	1 (2.3)	Dental problems	70	17 (24.3)
*Transposition of the great arteries*	43	1 (2.3)	Dysphagia/feeding difficulties	70	14 (20)
Ventricular septal defect	43	17 (39.5)	Hepatomegaly	72	9 (12.5)
Atrial septal defect	43	3 (7.0)	**Kidney Anomaly**	72	14 (19.4)
Bicuspid aorta	43	3 (7.0)	**Musculoskeletal Anomaly**	72	13 (18.1)
Patent ductus arteriosus	43	2 (4.7)	**Eye Anomaly**	70	8 (11.4)
Pulmonary stenosis	43	1 (2.3)			
**Endocrine Findings**	69	25 (36.2)			
Short stature	66	21 (31.8)			
Hypocalcemia	69	21 (30.4)			
Hypothyroidism	54	6 (11.1)			

Serum immunoglobulin levels, lymphocyte counts, T and B cell subsets, and lymphocyte activation were all evaluated according to reference ranges for age [10–14]. We defined a hemoglobin level of less than 11 g/dl as anemia, an absolute neutrophil count of less than 1500/mm^3^ as neutropenia, a platelet count of less than 150000/mm^3^ as thrombocytopenia, a platelet size above 12 fl as macrothrombocyte, and an eosinophil count above 500/mm^3^ as eosinophilia [15, 16]. T cell subgroup analysis involved RTE (CD4+CD31+CD45RA+), naive T helper cells (Th) (CD4+CCR7+CD45RA+), central memory Th (CD4+CCR7+CD45RA-), effector memory T (TEM) helper (CD4+CCR7-CD45RA-), effector memory CD45RA+ (TEMRA) Th (CD4+CCR7-CD45RA+), naive T cytotoxic cells (Tc) (CD8+CCR7+CD45RA+), central memory Tc (CD8+CCR7+CD45RA-), effector memory Tc (CD8+CCR7-CD45RA-), and TEMRA Tc (CD8+CCR7-CD45RA+). B cell subgroup analysis involved naive B cells (CD38highIgMhigh), transitional B cells (CD19+CD27-IgD+), memory B cells (CD19+CD27+), switched memory B cells (CD19+CD27+IgD-), nonswitched memory/marginal zone-like B cells (CD19+CD27+IgD+), active B cells (CD19+CD38-CD21low), and plasmablasts (CD19+CD38highIgM-) [11, 14, 17]. Lymphocyte activation was assessed by measuring CD25 and CD69 expression through flow cytometry 48 hour after stimulating lymphocytes with phytohemagglutinin compared to healthy controls [18, 19].

## Statistics

We analyzed the data with IBM SPSS Statistics 23.0. We presented the clinical parameters, treatment approaches, and outcomes with descriptive statistics. Comparisons between two independent groups of numerical variables are tested with the Mann‒Whitney U test if parametric test assumptions are valid. We evaluated the relationships between categorical variables using the Pearson Chi-square test or Fischer`s Exact test based on the expected frequencies. We consider a p-value below 0.05 as statistically significant. Relationships between quantitative variables are analyzed using the Pearson correlation coefficient when parametric test assumptions are valid and the Spearman correlation coefficient when not valid.

## Results

### Genetic Findings

Sixty-six (91.7%) patients received a genetic diagnosis (Table S1), most diagnosed using fluorescence in situ hybridization (FISH) analysis, while some underwent multiplex ligation-dependent probe amplification and microarray analysis. No deletion was detected in four patients by FISH analysis. Another patient with no deletion by FISH analysis showed a 22q11.2 deletion by microarray. Genetic studies were not present in two patients.

### Demographical Characteristics

Three patients (P33, P38, P49) (Supplementary appendix) had DGS with congenital athymia, and 69 had DGS. The M/F ratio was 41/31 (1.3). The median age at diagnosis was 1.7 years (0 days −22 years). The follow-up period was 21.7 months (0 days-17.3 years), the diagnostic delay was 12 months (0 days −17 years), and the age of symptom onset was 18.2 days (0 day-10 years) (Table S1). During the follow-up period, six patients (8.3%) died.

### Clinical Findings

We present the clinical findings in Tables 1-S1, Figure S2, Figure S3, and Suppl. appendix. Commonly prompting the first visit to the hospital were cardiac reasons (30.6%, murmur, cutis marmoratus, etc.), neurological reasons (15.3%, seizure, intellectual disability, etc.), and failure to thrive (15.3%) (Figure S2A). Craniofacial and neuropsychiatric features account for approximately 97% and 86.1%, respectively. Patient’s 75.7% (53/70) of the patients had developmental delays, 65.7% (44/67) had special education status, 39.4% (28/71) had seizures, 52.4% (11/21) had central nervous system malformation, and 10 had attention deficit hyperactivity disorder (ADHD). Among the 28 patients with seizures, 11 also presented with hypocalcemia. Of the patients with seizures, 26 had electroencephalograms (EEG), and eight had revealed pathological findings, including epileptic activity, dysrhythmia, baseline disorders, or baseline wave slowing. Additionally, 14 out of the 28 patients (50%) were on antiepileptic drugs. Forty-three patients (61.4%) had congenital heart defects, with common defects being conotruncal cardiac anomalies (n = 17, 39.5%), such as tetralogy of Fallot, aortic arch anomalies, and ventricular septal defects. One-third of patients had hypocalcemia, and 11.1% had hypothyroidism. Thirteen (n = 21, 61.9%) patients with hypocalcemia had a cardiac anomaly, and nine had T-cell lymphopenia. Six out of nine patients with hypocalcemia, cardiac anomalies, and T-cell lymphopenia died. In our cohort, the mortality risk was about 2/3 in patients with hypocalcemia, cardiac anomaly, and lymphopenia (Figure S3).

About half (54.2%) of the patients had at least one problem related to the digestive system. Kidney anomalies were present in 14 (19.4%) patients. Thirteen patients (18.1%) had musculoskeletal findings.

### Immunological Findings

#### Complete Blood Counts, Serum Immunoglobulins, and Lymphocyte Subgroups

Lymphopenia, anemia, thrombocytopenia, eosinophilia, and neutropenia were found in 30 (41.7%), 16 (22.2%), 15 (20.8%), 10 (13.9%), and 1 (1.4%) patient(s), respectively. The immunological data are summarized in Table S2. When percentages are presented below, the denominator is the number of patients with available data.

**Fig. 1:**
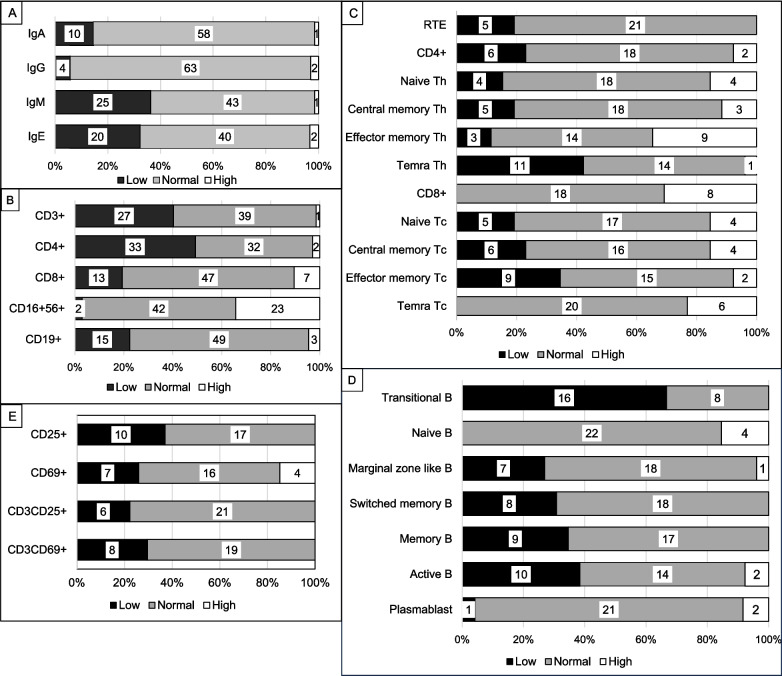
Distribution of immunological values according to age-matched reference ranges. **A**, immunoglobulin values. **B**, lymphocyte subset counts. **C**, T cell subgroup percentages. **D**, B cell subgroup percentages. **E**, lymphocyte activation tests. Patient numbers are shown in white boxes in the center of each colored box. For IgE; low < 5 IU/ml, high > 150 IU/ml. Th; CD4+ T helper cells. Tc; CD8+ T cytotoxic cells.

Serum Ig levels, evaluated according to age-matched references (Fig. 1A). Thirty-eight patients had normal/high IgA, IgG, and IgM values. No patient had panhypogammaglobulinemia. Low IgM (36.2%, n = 25), IgE (32.2%, *n* = 20), IgA (14.5%, *n* = 10), and IgG (5.8%, *n* = 4) levels were detected in the cohort. Two patients displayed elevated IgE values. Among patients with low IgA levels, six had low IgE levels. The association between low IgA and low IgE levels (below 5 IU/ml) was statistically significant (*p* = 0.011). Furthermore, strong correlations are present between IgG and IgA, as well as between IgG and IgE (r = 0.687, *p* < 0.001; r = 0.493, *p* < 0.001, respectively).

Positive anti-HBs titers (indicating a protein antibody response) were observed in 33 (*n* = 33/55, 60%) patients. Isohemagglutinin responses, assessed using anti-A and anti-B titrations, were normal (above 1/16, *n* = 26).

Sixty-seven patients'lymphocyte subset counts according to age-matched reference ranges are present in Fig. 1B. CD3+ and CD4+ cell counts were low in 27 (40.3%) and 33 (49.3%) patients, respectively. B lymphocytes (*n* = 49, 73.1%), CD8+ T cells (*n* = 47, 70.1%), and NK cells (*n* = 42, 62.7%) were within normal levels in the given number of patients. While the NK cell percentage was high in 38 patients (56.7%), the NK cell count was high in 23 patients (34.3%) (Figure S4). The median ratio of CD4+ lymphocytes to CD8+ lymphocytes was 1.17 (0–5.90).

A weak correlation was found between CD3+ T cell and NK cell counts (r = 0.256, *p* = 0.036). However, a strong correlation was present between CD8+ T cell and NK cell counts (r = 0.358, *p* = 0.003). Interestingly, a strong correlation was recorded between CD8+ T cell and B cell counts (r = 0.642, *p* < 0.001) and between T and B cell counts (r = 0.611 and *p* < 0.001). We assessed the correlation between the CD4+ T cell counts and immunoglobulin levels and found no significant correlation (Table S2).

Additionally, 15 patients` IgA, G, M levels, and lymphocyte subset counts were normal/high according to the age-matched reference range (seven patients had T and B cell subset percentages, these patients displayed distributions different from normal; six patients had lymphocyte activation test, three of them had low activation response).

#### T cell Subgroup Analysis

**Fig. 2:**
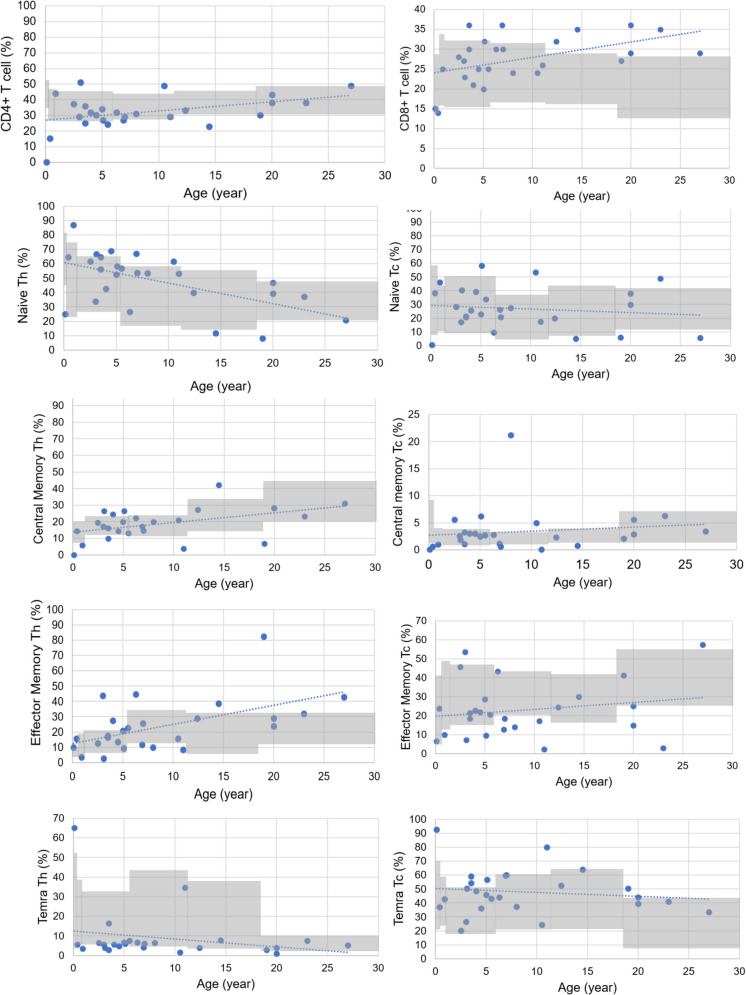

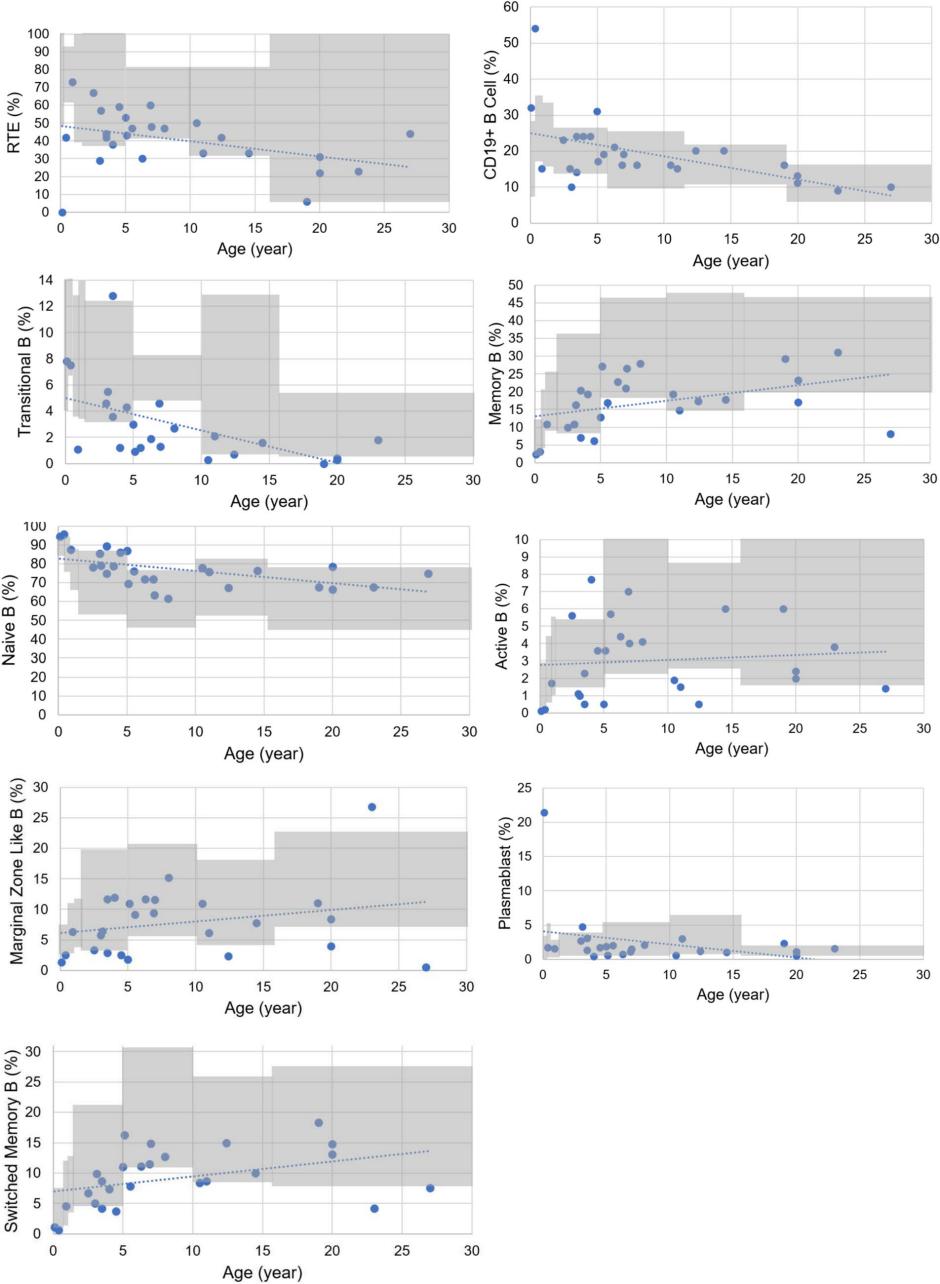
Patients` T and B cell subgroup results. The gray areas indicate the normal age-matched reference ranges. The dotted line represents the trend with age in the results of DGS patients. T and B cell subgroup data are available for 26 patients. Th; CD4 + T helper cells. Tc; CD8 + T cytotoxic cells.

The T-cell subgroup percentages for 26 patients are presented in Table S2, Fig. 1C, and Fig. 2. The RTE percentages were within age-matched reference ranges in 21 (80.8%) patients and low in 5 (19.2%).

According to the age-matched references, the CD8+ T cell counts were normal or high in 80% of patients. Remarkably, no patient had a low percentage of CD8+ TEMRA cells. However, the percentages of CD4+ TEMRA cells were low in 42.3%(11/26) of patients.

In 19.2% of our DGS patients, the RTE cell percentages were lower than the age-matched reference ranges (Figs. 1 and 2). The percentage decrease in RTE cells with age was also prominent (r = −0.314, *p* = 0.118), although not statistically significant. Similarly, the naive CD4+ percentage decreased with age (r = −0.491, *p* = 0.011). The central memory CD4+ and effector memory CD4+ percentages increased (r = 0.530, *p* = 0.005; r = 0.508, *p* = 0.008), but the percentages of CD4+ TEMRA cells and the percentages of respective CD8+ cell subgroups showed no significant changes with age (Table S2).

An inverse relationship was present between the naive CD4+ cell percentages and the IgG levels (r =—0.453, *p* = 0.020), and there was a positive correlation between the central memory CD8+ and effector memory CD8+ cell percentages with IgG levels (r = 0.674, p < 0.001 and r = 0.463, *p* < 0.001, respectively).

A strong correlation was present between the RTE cell percentage and the CD3+ cell count (r = 0.678 and *p* < 0.001); as expected, this relationship was stronger for CD4+ lymphocytes (r = 0.719, *p* < 0.001) than for CD8+ lymphocytes (r = 0.404 and *p* < 0.04). Additionally, RTE percentages correlated with the CD19+ cell counts (r = 0.400 and *p* < 0.043).

Twenty (95.2%) patients out of 21 whose RTE cell percentages were within age-matched references had their serum IgA levels within age-matched references. Five patients had a low RTE cell percentage, and three (60%) had low IgA levels. A statistically significant correlation was present between the RTE cell percentages and the serum IgA levels (p = 0.014, tested with Fisher's Exact test). An inverse correlation was present between the RTE cell percentages and the IgM levels (r = −0.448, p = 0.022).

#### B Cell Subgroup Analysis

The results for the B-cell subgroup percentages are in Table S2, Fig. 1D, and Fig. 2. In 26.9%, 30.8%, and 38.5% of patients, the percentages of marginal zone, switched memory, and active B cells were low, respectively, while 67% of patients had low transitional B cell percentages. Plasmablasts and naive B cell percentages were within the age-matched reference ranges or high in almost all patients. There was no patient with a high memory B cell percentage.

When evaluated at different age groups among the patients, the percentages of naive B, transitional, and plasmablast cells decreased with age (r = −0.660, *p* < 0.001; r = −0.669, *p* < 0.001; r = −0.393, *p* = 0.058, respectively). Memory B and switched-memory B cell percentages increased, and the marginal zone-like and active B-cell percentages did not change with age (Table S2).

We evaluated the relationship between B-cell subgroup percentages and serum immunoglobulin levels. The memory B-cell percentages and serum IgM levels were low in DGS patients, showing a positive correlation (r = 0.496, *p* = 0.01). The percentages of switched-memory B cells correlated with serum IgG and IgM levels (r = 0.402, *p* = 0.042; and r = 0.518, *p* = 0.007, respectively). Additionally, the percentages of marginal zone B cells correlated with serum IgM levels (r = 0.412, *p* = 0.037). Notably, there was an inverse relationship between the transitional B-cell percentage and serum IgG and IgM levels (r = −0.457, *p* = 0.025; r = −0.538, *p* = 0.001).

#### Lymphocyte Activation Test

CD25 expression on CD3+ T cells was low in 6 (22.2%) of the patients (n = 27), and CD69 expression on CD3+ T cells was low in 8 (29.6%) when stimulated with phytohemagglutinin (Fig. 1E). A low IgA level was associated with low lymphocyte activation (p<0.05) (Table S2).

#### Infections and Pathogens

The median number of infections per year in patients (n = 67) was four (2–10). The most common presentation was pneumonia (52.8%), followed by otitis (30.6%), sinusitis (16.7%), and sepsis (15.3%). The microorganisms evaluated to be causative during infectious diseases were *Klebsiella pneumoniae* (n = 9) and *Staphylococcus epidermidis* (n = 3). Sixty (83.3%) patients experienced at least one hospitalization due to infection in their lifetime.

#### Treatment

Twenty-eight (38.9%) patients were on antibacterial prophylaxis, and two were on antifungal prophylaxis (Table S1). Additionally, six patients were on immunoglobulin therapy, two with ITP and four with hypogammaglobulinemia.

Thymus transplantation was the plan for one of the three DGS with congenital athymia, and hematopoietic stem cell transplantation was considered for the other patient (the thymus transplantation wasn’t possible at that time). Unfortunately, both patients died before therapy.

## Discussion

The typical triad of 22q11.2 deletions syndrome is congenital heart disease, immunodeficiency, and hypocalcemia [3, 5]. In DGS patients, chromosomal deletions develop spontaneously, and 5–10% are autosomal dominant [4]. In our study, the low percentage of parents with 22q11.2 deletions syndrome may suggest the probability of undiagnosed parents. Poirsier et al. showed that the ratio of boys to girls was 0.87 [20]. Our study showed an M/F ratio of 1.3. Ninety-seven percent of the present cohort had at least one craniofacial dysmorphic finding, and about three-quarters had neurodevelopmental delay. According to another case series, 70% of patients had a delay in speech and language, and 53% had a psychomotor delay [21]. In 2 series, 64% to 79% of patients have conotruncal congenital heart disease, respectively [21, 22]. We found a similar ratio of congenital heart diseases (61.4%), and 22q11.2 DS/DTD should be considered in patients in the presence of cardiac findings [6, 20, 22].

In the present cohort, pneumonia (52.8%), otitis (30.6%), sinusitis (16.7%), and sepsis (15.3%) were common. In contrast, upper respiratory infections (sinusitis (6-27%), otitis (22-25%), and lower respiratory tract infections (bronchitis (7-20%) and pneumonia (4-20%)) were reported in other series [21, 23].

Hypocalcemia is observed in 14.5–55% of DGS patients [4, 20]. Increased mortality was reported in 22q11.2 DS patients with cardiac anomalies and hypocalcemia [24–26], which is in line with the data of our cohort, where the mortality risk was about 2/3 in patients with hypocalcemia, cardiac anomaly, and lymphopenia.

Due to the chromosomal deletion region in DGS, concomitant macrothrombocytopenia and bleeding susceptibility may be present. Ninety percent of patients with 22q11 deletion have a deletion in the glycoprotein Ib platelet-subunit-beta gene region, where homozygous mutations cause Bernard Soulier syndrome [27]. Additionally, the ITP frequency may increase up to 200-fold [27]. In our series, two ITP patients were present.

Less than 1.5% of DGS patients with congenital athymia are reported [28]. However, our center records a relatively high rate (4.2%), possibly related to its referral center status.

In this study, low IgE levels correlated with low IgA levels when evaluated according to age-matched references. Lawrence et al. suggested a low serum IgE (below 5 < IU/ml) as a marker with high sensitivity and specificity for common variable immunodeficiency (CVID) [29]. Our study is the first to show low IgE levels in DGS patients. Low IgE levels and IgA levels are likely due to impaired T-cell help.

In this study, DGS patients had generally high/normal-ranged B and NK cell counts and low T-cell counts. The increase in NK cell number may compensate for the decrease in thymus-derived T cells. T-cell counts are low in the first years of life, normalizing over time. Peripheral T-cell homeostatic proliferation compensates for the thymic involution-related decrease in T-cell production in 22q11.2 DS patients [30]. As thymic production is limited, the only available avenue is the proliferation of existing T cells, expressed by conversion of the naive surface phenotype (CD4+CD45RA+) to the memory phenotype (CD4+CD45RO+) [30]. Therefore, the naive phenotype decreases, whereas the memory phenotype increases in children with 22q11.2 deletion, as shown in the present study [4, 30, 31]. Central memory cells, the main proliferative subgroup that responds strongly to antigens and cytokines, are expected to decrease in patients with 22q11.2 DS [4, 32, 33]. In our study, one-third of the patients had low CD8+ effector memory cells, and about 10% had low CD4+ effector cells. In 80% of the patients, the CD8 + cell counts, and in all patients, the CD8+TEMRA cell percentages were in the normal range or high. These findings suggest that CD8 TEMRA cells account for a definite proportion of CD8+ cells, and CD8+ central memory to TEMRA cell differentiation may be critical in DGS patients. CD8+TEMRA cells represent terminally differentiated effector cells associated with protracted antigen exposure and show immunosenescence [34]. CD8+TEMRA cells may resemble effector memory CD8+ T cells and may have an increased expression of molecules associated with terminal differentiation and cytotoxicity, such as PR56, granzyme B, perforin, CD244, and KLRG1. CD8+TEMRA cells play a critical role against viral infections by killing infected cells [35, 36]. In patients with 22q11.2 DS, prolonged viral infections are common, and velopharyngeal incompetence likely contributes to the risk of upper airway infection [37]. The accumulation or the peripheral homeostatic proliferation of CD8+TEMRA cells in this DGS patient cohort is probably due to recurrent infections.

As stated above, one-third of the patients had low CD8+ effector memory cells, and 10% had low CD4+ effector cells. Several factors may play a role in the differences observed in the differentiation of CD4+ and CD8+ effector memory cells. The time of antigen exposure required to launch the proliferative program for naive CD8+ T cells seems to be less than that needed for naive CD4+ T cells [38–42]. Also, CD8+ T cells divide sooner and have a faster rate of cell division than CD4+ T cells [38, 43–45]. Since naive CD8+ T cells more readily develop into effector cells after short-term primary stimulation compared to CD4+ T cells, there are differences in the dispersion of the CD4+ and CD8+ subgroups (Fig. 2).

Recent thymic emigrant cells increase in the first months of life, decrease after 9–15 months, and then decrease with age like naive CD4+ T cells [13, 46]. Recent thymic emigrant cells indicate thymic production that decreases over time in healthy people and DTD/DGS patients [46]. However, the decrease in RTE percentage seems to be enhanced with age in DGS patients in our study, although not statistically significant. The RTE percentages correlate with serum IgA level, and low IgA level has been associated with impaired T-cell function in the present study. Thus, IgA levels may provide information about the immune status of DGS patients besides RTE cells. The RTE cell percentages and serum IgA levels were lower than the age-appropriate reference ranges in 19.2% and 14.5% of our DGS patients, respectively (Fig. 1).

Hypogammaglobulinemia develops in a small number of 22q11.2 DS/DTD patients, but almost all exhibit humoral dysfunction and B-cell maturation defects since T-cell help is essential for B-cell differentiation and proliferation [4]. The number of naive B cells increased, while the number of switched memory B cells decreased in 22q11.2 DS patients in a cohort described by Derfalvi et al. [8]. In the present study, naïve B cells of patients were in the normal range or high, and one-third of memory and switched memory B cells were low. Two-thirds of patients in our cohort had low transitional cell percentages. The low transitional B-cell percentages state an early B-cell developmental stage defect characterized by accumulations of precursors [8]. Increased conversion from active B cells to plasmablasts might have occurred as a compensatory mechanism since the active B cell percentages were low while the plasmablast percentages were in normal ranges. We describe an increase in all memory B cell percentages with age in our cohort, while the transitional B cell, naive B cell, and plasmablast percentages decrease with age among our patient group. Transitional B cells and switched memory B cells correlated with IgM and IgG levels, and the correlation was stronger with IgM.

Immunoglobulin and T-cell proliferative responses are generally normal in the first years of life in most patients with 22q11.2 DS/DTD [23, 47, 48]. The mitogen response tends to decrease with age, although nonsignificant between age groups [23]. Low lymphocyte activation was described in three of six patients with 22q11.2 deletion and antibody deficiency [48], while Chinen et al. showed that the lymphoproliferative response to phytohemagglutinin is sufficient in DGS patients [47]. In the present cohort, CD3+CD25+ expression was low in one-fifth of the patients, and CD3+CD69+ expression was low in one-third of patients after PHA stimulation, which is consistent with a decreased response in a subgroup of patients. The polysaccharide antibody response was sufficient in all patients tested, and the protein antibody response is similar to the general population based on the response to hepatitis B vaccination based on two prior studies [49, 50].

The general treatment approach is supportive; 38.6% (n = 28) of the patients received antibiotic prophylaxis, and only six (9%) patients received IVIG treatment in this cohort. In a study including 228 patients, antibiotic prophylaxis and IVIG replacement therapy was implemented in 17% and 3%, respectively [21]. Patel et al. also reported the percentage of patients on IVIG treatment as 3% [5]. Mortality has been reported in 4% of patients with 22q11.2 DS [22]. Patients die mostly from cardiac causes in the first year of life [21]. In our study, the mortality rate was 8.3%.

Our study has several limitations. Due to its retrospective nature, standardized immunological, microbiological, pathological, and genetic evaluation could not be uniform in each patient. Only a subset of patients (n = 26) underwent T/B-cell subgroup analyses. As there is no follow-up measurement in the study, our conclusions regarding changes over time are derived from patient data and age-matched references since the patients presented are of different ages. Repeated measurements would be better. The age-matched data may vary in similar populations and is affected by some factors (early exposure to infectious, environmental pathogens, and socioeconomic level), which possibly cause a decrease in naive cells and an increase in effector memory and active cells [51–57]. The references for T/B cell subgroup analyses are population-specific, but the analyses were standardized using the same reference tables for all patients [10–14]. Additionally, patients without 22q11.2 deletion did not undergo thymic imaging despite having clinical findings consistent with a thymic defect. A thymic ultrasound may be added to the criteria for thymic developmental defects.

Normal range or high NK, CD8+ lymphocyte counts, and CD8+ TEMRA cell percentages may suggest peripheral homeostatic proliferation due to recurrent viral infections. The correlation between low RTE cell percentages, low serum IgA levels, and impaired T-cell function sheds light on the immune status of DGS patients. DGS patients with hypocalcemia, congenital cardiac anomaly, and lymphopenia exhibit a more severe disease with an increased mortality risk. Once a patient is diagnosed with DGS, detailed immunological evaluation and a multidisciplinary approach are crucial, ensuring timely interventions to maximize the quality of life.

## Supplementary Information

Below is the link to the electronic supplementary material.

Supplementary file1 DiGeorge syndrome patients with congenital athymia and clinical findings (DGS patients with and without congenital athymia) (DOCX 19.2 KB)

Supplementary file2 Patients included in the study (PPTX 37.8 KB)

Supplementary file3 A. Symptoms leading to initial presentation. B. Common systemic features associated with DiGeorge Syndrome (the number of patients is given in Table 1) (Adobe Stock image (#341535919)) (PPTX 254 KB)

Supplementary file4 Presence of lymphopenia and cardiac malformation in DGS patients with hypocalcemia (DOCX 44.8 KB)

Supplementary file5 Distribution of lymphocyte subset percentage according to age-matched reference ranges (PPTX 40.5 KB)

Supplementary file6 Demographical characteristics and treatment of patients with 22q11.2 deletion syndrome/DTD, and genetic evaluation and complete blood count, immunoglobulin level, lymphocyte, T cell and B cell subgroup, lymphocyte activation test analysis of patients with 22q11.2 deletion syndrome/DTD (DOCX 27.1 KB)

## Data Availability

On a reasonable request, the data supporting study’s findings are available from the corresponding author.

## Abbreviations

DGS: DiGeorge Syndrome
DTD: Defect in thymic development
DS: Deletion syndrome
ESID: European Society of Immunodeficiency
FISH: Fluorescence in situ hybridization
MLPA: Multiplex ligation-dependent probe amplification
RTE: Recent thymic emigrant
TEM: T effector memory CD45RA-
TEMRA: T effector memory CD45RA+
