# Supplementary material for: Clinical and Immunological Features of a Large DiGeorge Syndrome Cohort
Source: J Clin Immunol. 2025 Jun 3;45(1):103. doi: 10.1007/s10875-025-01884-0 (PMC12133924; doi:10.1007/s10875-025-01884-0)
Supplement: Supplementary file 1 — Supplementary file1 DiGeorge syndrome patients with congenital athymia and clinical findings (DGS patients with and without congenital athymia) (DOCX 19.2 KB) [file 10875_2025_1884_MOESM1_ESM.docx]

**DiGeorge Syndrome Patients with Congenital Athymia**

P33 was referred to the immunology clinic when she was one month old. The lymphocyte count was 5800/mm^3^, the IgA value was less than 6.7 mg/dL, the CD3 cell count was 0/mm^3^, and the percentage of RTE cells was 0. In addition, the patient had a VSD and hypocalcemia. Thymus transplantation was planned but could not be performed due to CMV viremia. She died at 8 months of age due to sepsis. During the follow-up period, the patient's mother was also diagnosed with 22q11.2 deletion syndrome. There was consanguinity between the parents of P33, and the uncle of P33 had Bruton’s agammaglobulinemia.

P38 was referred to our hospital when he was 2.5 months old. The patient had a history of hypocalcemic seizures and sepsis. Echocardiography revealed VSD and ASD. The lymphocyte count was 1100/mm^3^, the IgA value was low, and the CD3 cell count was 73/mm^3^. T-cell subgroup evaluation was not possible at our hospital at that time. During follow-up, a skin rash and hepatosplenomegaly developed. She could not undergo hematopoietic stem cell transplantation because of her poor general condition (the thymus transplantation wasn’t possible at that time). The patient died of sepsis and heart failure at six months of age.

P49 was born in our hospital and was followed in the neonatal department due to the tetralogy of Fallot that was noticed in the prenatal period. He had hypocalcemia, and the lymphocyte count was 600/mm^3^. Together with the severe lymphopenia and hypocalcemia, he had congenital heart disease [9]. The 22q11.2 deletion was detected via FISH analysis. Unfortunately, the patient did not have immunoglobulin measurements or lymphocyte subgroup analysis. The patient died at the age of 10 days due to heart failure and sepsis.

**Clinical Findings (DGS Patients with and without Congenital Athymia)**

**Craniofacial and Neuropsychiatric Features:**Characteristic craniofacial findings associated with DGS/DTD were present in 64 of 66 (97%) patients; 97% (n=64) of them had at least one dysmorphic and 80% (n=53) had at least two dysmorphic features. Frequent findings were auricular deformity, micro/retrognathia, and ocular abnormalities. A cleft palate was present in 23.9%, and a cleft lip anomaly in 2.8% of the patients. Hearing loss was found in 10 of 70 (14.2%) patients. Eight patients (11.4%) had eye anomalies such as strabismus, myopia, sclerocornea, and corneal opacity. 75.5% (n=53) of patients had developmental delays, 65.7% (n=44) had special education status, 39.4% (n=28) had seizures, %15.6 (n=10) had attention deficit hyperactivity disorder (ADHD), **11** of 21 patients (52.4%) had central nervous system malformation (hydrocephalus, cavum septum pellucidum variation, polymicrogyria, cerebral and cerebellar atrophy, thin pituitary gland, corpus callosum dysgenesis, etc.). EEG results were present in 26 patients with seizure history; eight had pathological findings like epileptic activity, dysrhythmia, baseline disorders, or baseline wave slowing. Fourteen of 28 (50%) patients were on antiepileptic drugs.

**Cardiac Findings:**Forty-one of 72 (56.9%) patients had murmurs on physical examination, and 18 of 57 patients (31.6%) had cardiomegaly on chest X-ray. Forty-three of 70 patients (61.4%) had a congenital cardiovascular defect (Table 1). Common major cardiac defects were conotruncal cardiac anomaly (39.5%; Fallot tetralogy, aortic arch anomalies, etc.) and ventricular septal defect (39.5%). There was no significant difference in age at diagnosis between patients with and without congenital cardiac anomaly (Mann-Whitney U, p=0.885).

Ten patients were on medical treatments like antihypertensives and diuretics. Catheter angiography and cardiac surgery were performed on 25 of 70 (35.7%) and 27 of 72 patients (35.7%), respectively. Four patients died due to cardiac anomalies (2 with tetralogy of Fallot, two with interrupted aortic arch).

**Endocrine Findings:**One-third of patient heights were below the 3rd percentile, and 40.9% were below the 5th percentile. One-third of patients had hypocalcemia, and 11.1% had hypothyroidism. Thirteen (61.9%) patients with hypocalcemia had a cardiac anomaly, and nine had T-cell lymphopenia.**All nine patients with hypocalcemia, cardiac anomaly, and T-cell lymphopenia died.** The mortality rate was 28.6% in patients with hypocalcemia, about 3.5 times the mortality in all patients (8.3%). Hypocalcemia is a poor prognostic factor for survival in the study group (p<0.001, with Fisher's Exact Test). Two patients were followed and treated by the endocrinology department for growth hormone deficiency.

**Other Systemic Findings:**Table 1 shows other systemic findings. Half of the patients had at least one problem related to the digestive system; 29.9% of patients had body weight below the 3rd percentile, 24.3% had dental problems (dental caries, shape, number anomalies, etc.), 20% had a swallowing dysfunction and/or feeding difficulties, two patients had gastrostomy. Anteverted anus, diaphragmatic hernia, esophageal atresia, cecum perforation, and volvulus were other gastrointestinal anomalies in the patients. Kidney anomalies were present in 14 (19.4%) patients, hydronephrosis in 7, nephrocalcinosis in 2, increased echogenicity in the renal parenchyma in 2, atrophic left kidney in 1, renal agenesis in 1, and multicystic dysplastic kidney in 1 patient. Thirteen patients (18.1%) had musculoskeletal findings. Scoliosis (46.1%) was common. Pes-planus, Sprengel deformity, cubitus varus, and clinodactyly were other findings. No patients had oncological/hematological malignancies.
