# Supplementary material for: Clinical and Immunological Features of a Large DiGeorge Syndrome Cohort
Source: J Clin Immunol. 2025 Jun 3;45(1):103. doi: 10.1007/s10875-025-01884-0 (PMC12133924; doi:10.1007/s10875-025-01884-0)
Supplement: Supplementary file 2 — Supplementary file2 Patients included in the study (PPTX 37.8 KB) [file 10875_2025_1884_MOESM2_ESM.pptx]

## Slide 1
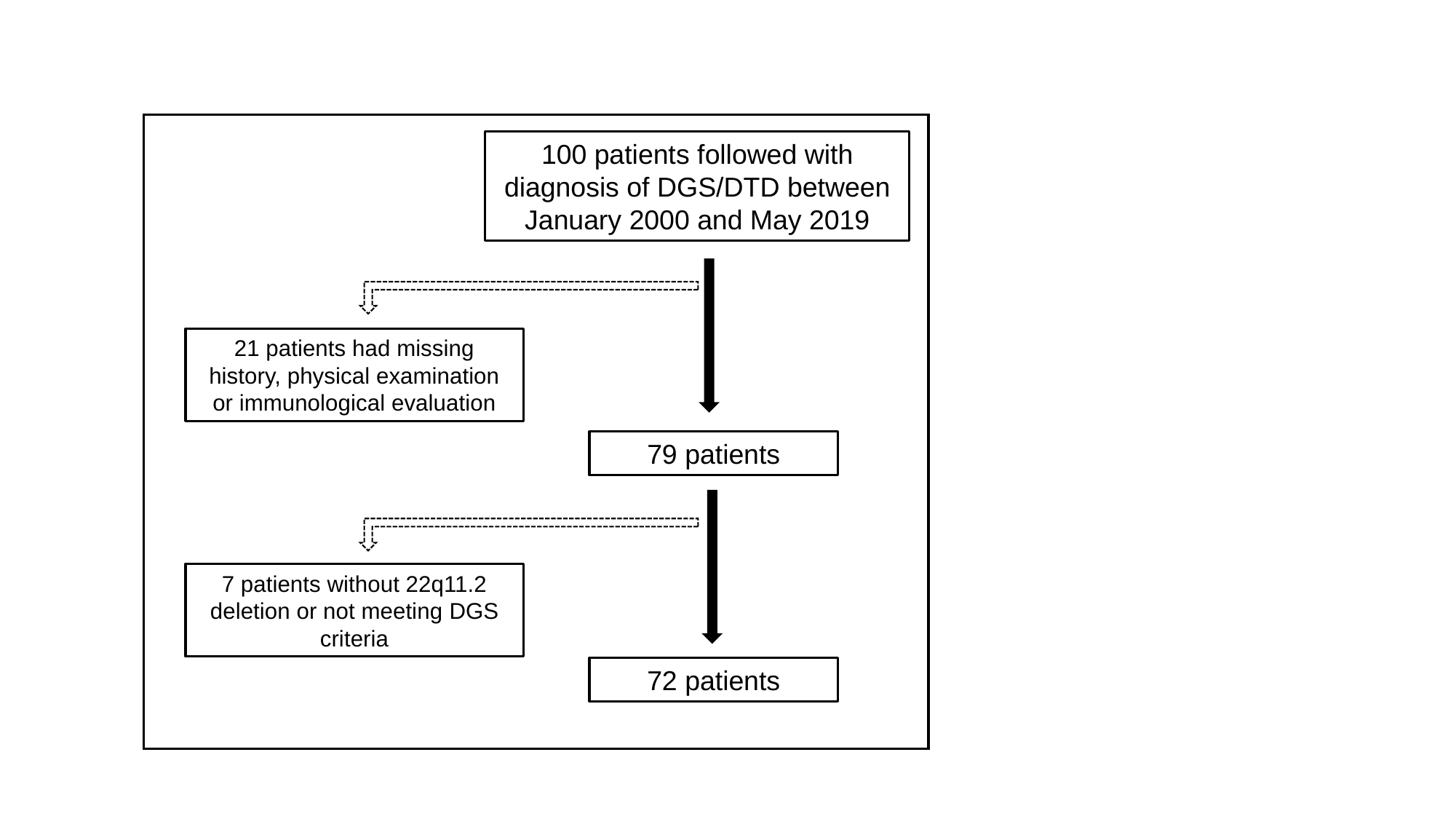

100 patients followed with diagnosis of DGS/DTD between January 2000 and May 2019
21 patients had missing history, physical examination or immunological evaluation
79 patients
7 patients without 22q11.2 deletion or not meeting DGS criteria
72 patients
