# Supplementary figures and images for: Clinical and Immunological Features of a Large DiGeorge Syndrome Cohort
Source: J Clin Immunol. 2025 Jun 3;45(1):103. doi: 10.1007/s10875-025-01884-0 (PMC12133924; doi:10.1007/s10875-025-01884-0)

## Slide 1
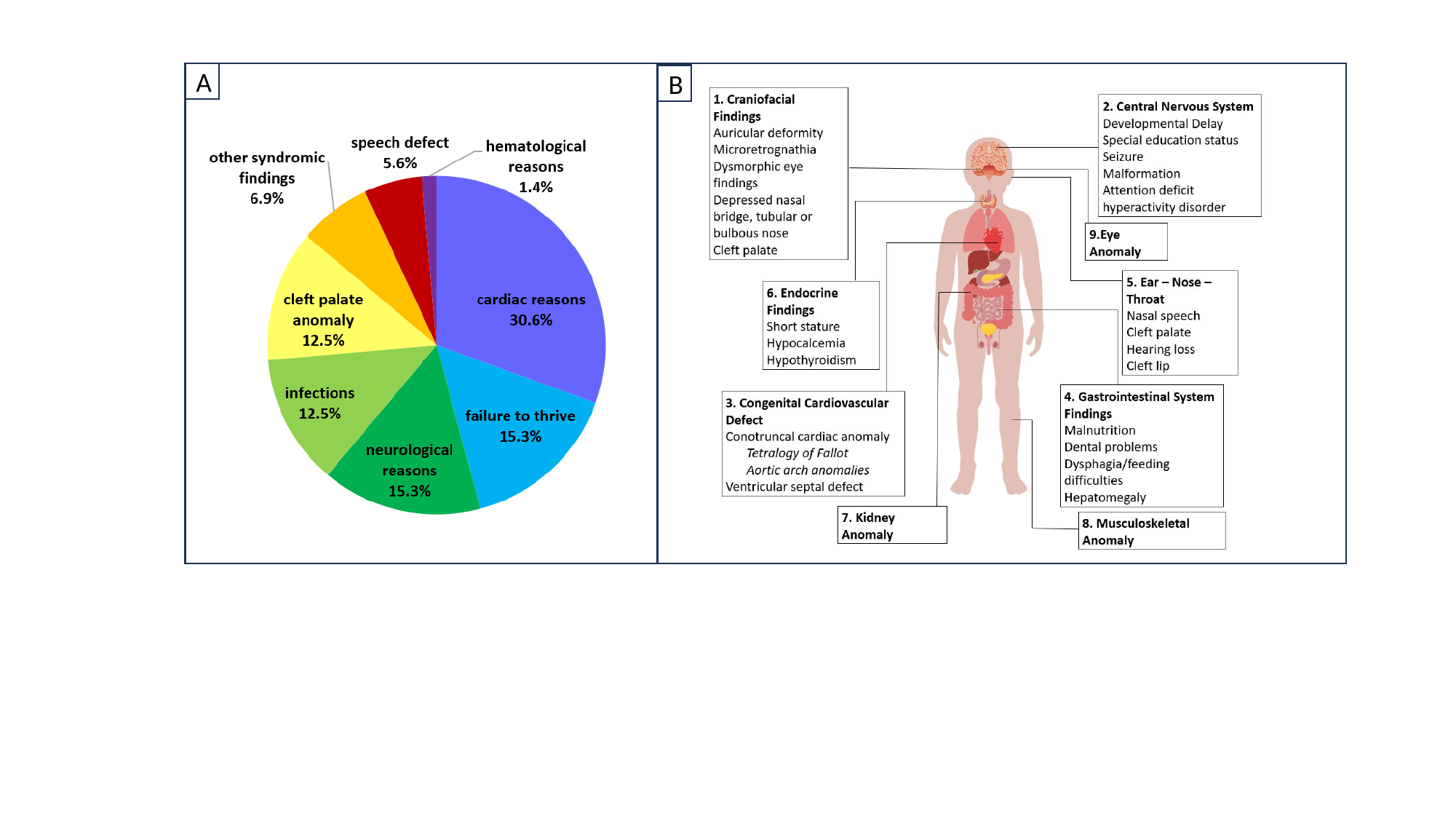

A
B

Supplement: Supplementary file 3 — Supplementary file3 A. Symptoms leading to initial presentation. B. Common systemic features associated with DiGeorge Syndrome (the number of patients is given in Table 1) (Adobe Stock image (#341535919)) (PPTX 254 KB) [file 10875_2025_1884_MOESM3_ESM.pptx]

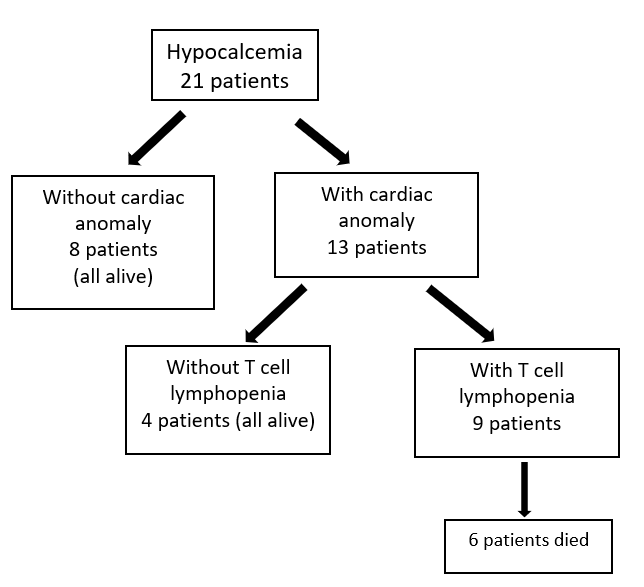

Supplement: Supplementary file 4 — Supplementary file4 Presence of lymphopenia and cardiac malformation in DGS patients with hypocalcemia (DOCX 44.8 KB) [file 10875_2025_1884_MOESM4_ESM.docx]

## Slide 1
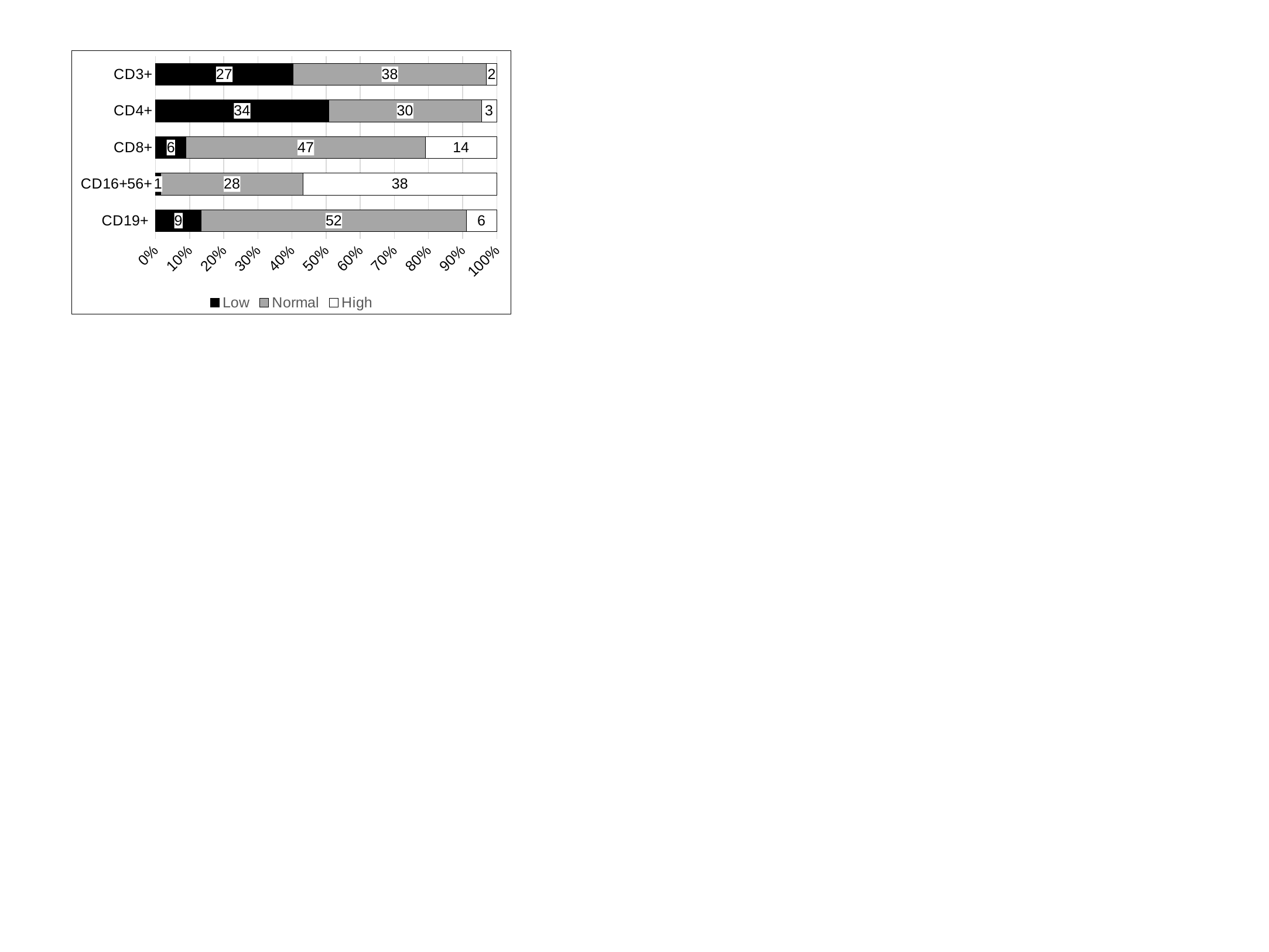

### Chart
| Category | Low | Normal | High |
|---|---|---|---|
| CD19+ | 9.0 | 52.0 | 6.0 |
| CD16+56+ | 1.0 | 28.0 | 38.0 |
| CD8+ | 6.0 | 47.0 | 14.0 |
| CD4+ | 34.0 | 30.0 | 3.0 |
| CD3+ | 27.0 | 38.0 | 2.0 |

Supplement: Supplementary file 5 — Supplementary file5 Distribution of lymphocyte subset percentage according to age-matched reference ranges (PPTX 40.5 KB) [file 10875_2025_1884_MOESM5_ESM.pptx]
