# Supplementary material for: Clinical and Immunological Features of a Large DiGeorge Syndrome Cohort
Source: J Clin Immunol. 2025 Jun 3;45(1):103. doi: 10.1007/s10875-025-01884-0 (PMC12133924; doi:10.1007/s10875-025-01884-0)
Supplement: Supplementary file 6 — Supplementary file6 Demographical characteristics and treatment of patients with 22q11.2 deletion syndrome/DTD, and genetic evaluation and complete blood count, immunoglobulin level, lymphocyte, T cell and B cell subgroup, lymphocyte activation test analysis of patients with 22q11.2 deletion syndrome/DTD (DOCX 27.1 KB) [file 10875_2025_1884_MOESM6_ESM.docx]

**Table S1.** Demographical characteristics, treatment of patients with 22q11.2 deletion syndrome/DTD, and genetic evaluation.

| **Demographical characteristics** | |
| --- | --- |
| **Sex** | **n (%)** |
| Female | 31 (43.1%) |
| Male | 41 (56.9%) |
| **Survival** | **n (%)** |
| Alive | 66 (91.7%) |
| Dead | 6 (8.3%) |
| **Relative with DGS^1^** | 5 (6.9%) |
| **Age** | **median (min-max)** |
| Alive (n=66) | 8.86 years (7 months-26 years) |
| Dead (n=6) | 6 month (11 days-2.5 years) |
| **Age of symptom onset** | 18.2 days (0 day-10 years) |
| **Age of diagnosis^2^** | 1.7 years (0 day-22 years) |
| **Diagnostic delay** | 12 months (0 day-17 years) |
| **Follow-up time** | 21.7 months (0 days-17.3 year) |
| **Treatment** | **n (%)** |
| **Antimicrobial prophylaxis** |  |
| Antibacterial prophylaxis | 28 (38.9**%**) |
| Antifungal prophylaxis | 2 (2.8**%**) |
| IVIG (n=66) | 6 (9**%**) |
| **Genetic Test (+)^3^** | **66 (91.7%)** |
| FISH (+)^4^ | 55 (76.3**%**) |
| Microarray^5^ (+) | 6 (8.3**%**) |
| *FISH (+), MLPA (+)* | *3 (4.2***%***)* |
| *FISH (+), microarray (+)* | *1 (1.4***%***)* |
| *FISH (-)*^6^*, microarray (+)* | *1 (1.4***%***)* |
| **Genetic Test (-)**^6^ | **4 (5.6%)** |
| FISH (-) | 4 (5.6**%**) |

^1^ The mothers of P16 and P33 had 22q11.2 deletion syndrome, P42 had a monozygotic twin sibling who was followed in another center, and P46 and P47 were monozygotic twins.

^2^ The earliest diagnosis was made by amniocentesis performed at the intrauterine 21^st^ week.

^3^ 22q11.2 deletion positive

^4^ Number of patients with only FISH analysis.

^5^ Number of patients with only microarray analysis.

^6^ 22q11.2 deletion negative.

**Table S2.** Complete blood count, immunoglobulin level, lymphocyte, T cell and B cell subgroup, lymphocyte activation test analysis of patients with 22q11.2 deletion syndrome/DTD.

| **Laboratory findings** | | |
| --- | --- | --- |
| **Complete blood count (n=72)** | **Median (min-max)** | |
| Hemoglobin (g/dl) | 12 (8.4-16.1) | |
| Leukocytes (/mm^3^) | 8300 (3700-19600) | |
| Absolute neutrophil count (/mm^3^) | 3650 (600-11600) | |
| Absolute lymphocyte count (/mm^3^) | 3200 (600-9700) | |
| Absolute eosinophil count (/mm^3^) | 200 (0-1700) | |
| Platelets (/mm^3^) | 207500 (35000-625000) | |
| Platelets (/mm^3^)^1^ | 210000 (102000-625000) | |
| Mean platelet volume^1^ (fL) | 9.4 (6.2-12.5) | |
| Mean platelet volume to platelet count ratio^1^ (MPV/PLTx10^5^) | 4.00 (1-10) | |
| Anemia (n/%) | 16 (22.2%) | |
| Neutropenia (n/%) | 1 (1.4%) | |
| Lymphopenia (n/%) | 30 (41.7%) | |
| Eosinophilia (n/%) | 10 (13.9%) | |
| Thrombocytopenia (n/%) | 15 (20.8%) | |
| **Immunoglobulins** | **Median (min-max)** | |
| IgA (mg/dl) (n=69) | 80 (<6.7-447) | |
| IgG (mg/dl) (n=69) | 978 (295-1890) | |
| IgM (mg/dl) (n=69) | 64.3 (4.2-302) | |
| IgE (IU/ml) (n=62) | 7.7 (0-1300) | |
| **Anti-HBs titers (n=55)** | | |
| Positive (n/%) | 33 (60%) | |
| Negative (n/%) | 22 (40%) | |
| **Isohemagglutinin titers (n=26)** | | |
| Positive (n/%) | 26 (100%) | |
| **Lymphocyte subgroups (n=67)** | **Median (min-max)** | |
| CD3+ (%) | 55 (0-84) | |
| (/mm^3^) | 1560 (0-6391) | |
| CD4+ (%) | 29 (0-59) | |
| (/mm^3^) | 850 (0-4150) | |
| CD8+ (%) | 26 (5-54) | |
| (/mm^3^) | 825 (149-2910) | |
| CD16+56+ (%) | 19 (1-71) | |
| (/mm^3^) | 513 (50-4118) | |
| CD19+ (%) | 20 (2-46) | |
| (/mm^3^) | 629 (26-2376) | |
| CD4+ / CD8+ (n=67) | 1.17 (0-5.90) | |
| **T cell subgroups percentages (n=26)** | **Median (min-max)** | |
| CD4+ | 31.5 (0-51) | |
| Naive Th | 53.25 (0-87) | |
| Central memory Th | 19.45 (0-41.9) | |
| Effector memory Th | 19.05 (2.7-82.3) | |
| TEMRA Th | 5.6 (1-65) | |
| TREC | 42.5 (0-73) | |
| CD8+ | 27.5 (14-36) | |
| Naive Tc | 25.85 (0.5-58.2) | |
| Central memory Tc | 2.65 (0.1-19.9) | |
| Effector memory Tc | 21 (2.3-57.3) | |
| TEMRA Tc | 45 (20.3-92.6) | |
| **B cell subgroup percentages (n=26)** | **Median (min-max)** | |
| CD19+ | 16.5 (9-54) | |
| CD20+ | 16.0 (10-54) | |
| Transitional (n=24) | 1.85 (0-12.8) | |
| Naive B | 76.25 (61.6-95.7) | |
| Marginal zone like B | 7.1 (0.5-26.8) | |
| Switched memory B | 8.7 (0.6-18.3) | |
| Memory B | 17.2 (2.4-31) | |
| Active B | 2.35 (0.1-7.7) | |
| Plasmablast (n=24) | 1.6 (0.4-41.40) | |
| **Lymphocyte activation tests (n=27)** | **Median (min-max)** | |
| CD25 expression | 76 (4-96) | |
| CD69 expression | 83 (13-94) | |
| CD3/CD25 expression | 56 (0-79) | |
| CD3/CD69 expression | 54 (1-80) | |
| **Correlations** | **r value** | **p value** |
| IgA and IgE with Fisher's Exact test |  | **P=0.011** |
| IgG and IgA with Spearman | r=0.687 | **p<0.001** |
| IgG and IgE with Spearman | r=0.493 | **p<0.001** |
| CD3+ T and NK cell counts  with Spearman | r=0.256 | **p=0.036** |
| CD8+ T cell and NK cell counts  with Spearman | r=0.358 | **p=0.003** |
| CD8+ T cell and CD19+ B cell counts  with Spearman | r=0.642 | **p<0.001** |
| CD3+ T cells and B lymphocyte counts  with Spearman | r=0.611 | **p<0.001** |
| CD4+ T-cell count and IgA level  with Spearman | r=-0.169 | p=0.174 |
| with Fischer's Exact test |  | p=0.213 |
| CD4+ T-cell count and IgG level  with Spearman | r=-0.206 | p=0.097 |
| with Fischer's Exact test |  | p=0.838 |
| CD4+ T-cell count and IgM level with Spearman | r=-0.075 | p=0.552 |
| with Fischer's Exact test |  | p=0.165 |
| CD4+ T-cell count and IgE level with Spearman | r=0.055 | p=0.668 |
| with Fischer's Exact test |  | p=0.541 |
| RTE percentage and age with Spearman | r= -0.314 | p=0.118 |
| Naive CD4+ percentage and age with Spearman | r=-0.491 | **p=0.011** |
| Central memory CD4+ and age with Spearman | r=0.530 | **p=0.005** |
| Effector memory CD4+ percentage with Spearman and age | r=0.508 | **p=0.008** |
| CD4+ TEMRA percentage an age with Spearman | r=-0.155 | p=0.451 |
| Naive CD8+ percentage and age with Spearman | r=-0.106 | p=0.605 |
| Central memory CD8+ percentage and age with Spearman | r=0.312 | p=0.121 |
| Effector memory CD8+ percentage and age with Spearman | r=0.076 | p=0.713 |
| TEMRA CD8+ percentage and age with Spearman | r=0.002 | p=0.994 |
| Naive CD4+ percentage and the IgG level with Spearman | r=- 0.453 | **p=0.020** |
| Central memory CD8+ percentage and IgG level with Spearman | r=0.674 | **p<0.001** |
| Effector memory CD8+ percentage and IgG level with Spearman | r=0.463 | **p<0.001** |
| TEMRA CD4+ cell percentage and IgM level with Spearman | r=-0.404 | **p=0.041** |
| TEMRA CD4+ cell percentage and IgE level with Spearman | r=-0.415 | **p=0.035** |
| RTE percentage and the CD3+ cell count with Spearman | r=0.678 | **p<0.001** |
| RTE percentage and the CD4+ cell count with Spearman | r=0.719 | **p<0.001** |
| RTE percentage and the CD8+ cell count with Spearman | r=0.404 | **p<0.04** |
| RTE percentage and the CD19+ cell count with Spearman | r=0.400 | **p<0.043** |
| RTE percentages and CD16+56+ cell count with Spearman | r=0.227 | p=0.226 |
| RTE cell percentages and the serum IgA levels with Fisher's Exact test |  | **p=0.014** |
| RTE percentages and the IgM levels with Spearman | r=-0.448 | **p=0.022** |
| Naïve B cell percentage and age with Spearman | r=-0.660 | **p<0.001** |
| Transitional B cell percentage and age with Spearman | r=-0.669 | **p<0.001** |
| Plasmablast percentage and age with Spearman | r=-0.393 | p=0.058 |
| Memory B cell percentage and age with Spearman | r=0.565 | **p=0.003** |
| Switched-memory B cell percentage and age with Spearman | r=0.551 | **p=0.004** |
| Marginal zone-like B cell percentage and age with Spearman | r=0.241 | p=0.235 |
| Active B cell percentage and age with Spearman | r=0.311 | p=0.123 |
| Memory B-cell percentages and serum IgM levels with Spearman | r=0.496 | **p=0.01** |
| Switched-memory B cells percentage and IgG levels with Spearman | r=0.402 | **p=0.042** |
| Switched-memory B cells percentage and IgM levels with Spearman | r=0.518 | **p=0.007** |
| Marginal zone B cells percentage and IgM levels with Spearman | r=0.412 | **p=0.037** |
| Transitional B-cell percentage and IgG levels with Spearman | r=-.0457 | **p=0.025** |
| Transitional B-cell percentage and IgM levels with Spearman | r=-0.538 | **p=0.001** |
| IgA level and CD69 expression with Fisher's Exact test |  | p=0.054 |
| IgA level and CD3CD25 expression with Fisher's Exact test |  | **p=0.025** |
| IgA level and CD3CD69 expression with Fisher's Exact test |  | **p=0.004** |

^1^ Except for three patients with thrombocytopenia due to sepsis and ITP.

For %, denominator = number of patients with available data
